# Investigation of somatic mutation profiles and tumor evolution of primary oropharyngeal cancer and sequential lymph node metastases using multiregional whole‐exome sequencing

**DOI:** 10.1002/1878-0261.13407

**Published:** 2023-03-08

**Authors:** Nam Suk Sim, Su‐Jin Shin, Inho Park, Sun Och Yoon, Yoon Woo Koh, Se‐Heon Kim, Young Min Park

**Affiliations:** ^1^ Department of Otorhinolaryngology Yonsei University College of Medicine Seoul South Korea; ^2^ Department of Pathology, Gangnam Severance Hospital Yonsei University College of Medicine Seoul South Korea; ^3^ Center for Precision Medicine, Gangnam Severance Hospital Yonsei University College of Medicine Seoul South Korea; ^4^ Department of Pathology, Severance Hospital Yonsei University College of Medicine Seoul South Korea; ^5^ Department of Otorhinolaryngology, Gangnam Severance Hospital Yonsei University College of Medicine Seoul South Korea

**Keywords:** extranodal extension, intratumoral heterogeneity, lymph node metastasis, oropharyngeal cancer, WNT pathway

## Abstract

Lymph node (LN) metastasis is an important factor in determining the treatment and prognosis of oropharyngeal squamous cell carcinoma (OPSCC). Here, we compared the somatic mutational profiles and clonal evolution of primary and metastatic LNs using multiregion sequencing of human papilloma virus (HPV)‐positive OPSCC and HPV‐negative OPSCC. We performed high‐depth whole‐exome sequencing (200×) of 76 samples from 18 patients with OPSCC (10 HPV‐positive and 8 HPV‐negative), including 18 primary tumor samples, 40 metastatic LN samples, and 18 normal tissue samples. Among 40 metastatic LNs, 22 showed extranodal extension (ENE). Mutation profiles of HPV‐positive OPSCC and HPV‐negative OPSCC were similar to those reported previously. Somatic mutations in *CDKN2A* and *TP53* were frequently detected in HPV‐negative OPSCC. Somatic mutations in HPV‐positive OPSCC samples showed APOBEC‐related signatures. Somatic mutations from metastatic LNs showed a different pattern than the primary tumor. Somatic mutations acquired in the WNT pathway during metastasis showed a significant relationship with ENE. Clonal evolution analysis of primary and metastatic LNs showed that, in some cases, each metastatic LN originated from a different primary tumor sub‐clone.

## Graphical abstract

Metastatic lymph nodes (LNs) have unique somatic mutations compared with their primary tumor and to other metastatic LNs. Among these independent metastatic LNs, those with somatic mutations in the WNT pathway were correlated with the finding of extranodal extension (ENE). Each metastatic LN has an independent sub‐clone of origin, resulting in heterogeneity of metastatic LNs.

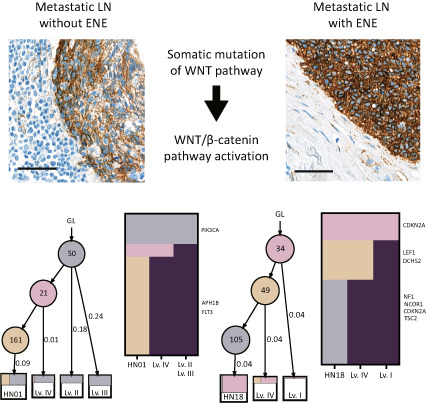

**Abbreviation**
AJCC: American Joint Cancer Committee
CADD: combined annotation‐dependent depletion
CNV: copy number variation
COSMIC: catalog of somatic mutations in cancer
DFS: disease‐free survival
ENE: extranodal extension
FFPE: formalin‐fixed paraffin‐embedded
GRCh38: genome Research Consortium human build 38
H&E: hematoxylin and eosin
HPV: human papilloma virus
LN: lymph node
MAF: minor allele frequency
NGS: next‐generation sequencing
OPSCC: oropharyngeal squamous cell carcinoma
OS: overall survival
SNP: single nucleotide polymorphism
TMB: tumor mutational burden
TSS: tumor‐specific survival
VAF: variant allelic frequency

## 1 Introduction

The incidence of oropharyngeal squamous cell carcinoma (OPSCC) associated with human papilloma virus (HPV) has increased in recent decades, whereas the incidence of OPSCC caused by smoking and drinking has decreased. HPV‐positive OPSCC has distinct clinical features compared with HPV‐negative OPSCC [1, 2, 3]. It occurs in relatively young patients and the response to treatment is favorable, so the patient's life expectancy is relatively longer than HPV‐negative OPSCC. Therefore, a range of clinical trials has investigated how to reduce treatment‐related morbidities and improve the quality of life of these patients [4, 5, 6, 7, 8]. However, despite the favorable prognosis of HPV‐positive OPSCC, the locoregional failure rate remains high at about 15%, and 10–20% of HPV‐positive OPSCC patients eventually die due to disease progression. Although HPV‐negative OPSCC due to smoking and drinking has decreased, it still accounts for 30% of OPSCC cases and the prognosis of these patients is worse than that of patients with HPV‐positive OPSCC. To improve the poor prognosis of HPV‐negative OPSCC patients and a subset of HPV‐positive OPSCC patients, molecular and genomic studies to understand the mechanisms underlying the progression and metastasis of OPSCC are required.

Most OPSCC cases originate from the palatine tonsils, with lymphatic metastasis occurring first in the level II area known as the first echelon lymph nodes (LNs). Thereafter, LN metastasis sequentially occurs to level III and level IV LNs along the jugular chain. During this process, extranodal extension (ENE), an adverse prognostic feature, occurs in some metastatic LNs. In the revised 8th American Joint Cancer Committee (AJCC) classification guidelines, the N classification was upgraded to N2a or N3b for the finding of ENE, including in HPV‐negative OPSCC [9, 10, 11, 12]. Although there is controversy surrounding the prognostic significance of ENE findings in HPV‐positive OPSCC, current NCCN guidelines recommend adjuvant treatment if ENE is observed.

Next‐generation sequencing (NGS) technology has made it possible to perform genomic analysis of cancer samples to identify genetic mutations related to the progression and metastasis of cancer in a cost‐effective manner. In this study, we performed multiregional whole‐exome sequencing of the primary tumor and metastatic LN samples to explore the genetic variation and tumor evolution of OPSCC tumors with sequential LN metastases and ENE. An understanding of the molecular genetic mechanisms underlying sequential lymph node metastasis and ENE will likely improve the treatment outcomes of OPSCC patients.

## 2 Materials and methods

### 2.1 Subject ascertainment

Among OPSCC patients who underwent surgery at Severance Hospital, 18 patients who had surgical specimens available and who provided written informed consent for use of these samples to obtain genetic information were enrolled in this study. An experienced specialized head and neck pathologist (S. J. Shin) marked the tumor‐corresponding region in formalin‐fixed paraffin‐embedded (FFPE) blocks through hematoxylin and eosin (H&E) staining. Tumor portions on LNs containing tumor metastasis were marked and matched with the primary tumor followed by manual dissection. For metastatic LN samples, metastatic LNs with ENE findings were preferentially collected, and if available, metastatic LNs samples were collected at different neck levels including II, III, and IV. Normal tissue samples were also obtained from surgical specimens. Clinical information was obtained from medical records. This study was approved by the Institutional Review Board of Yonsei University (4‐2020‐1370). All research procedures conformed to the principles of the Helsinki Declaration.

### 2.2 DNA extraction and library preparation

Genomic DNA was extracted from matched tumor, metastatic LN, and normal tissue samples that were re‐evaluated by a specialized head and neck pathologist using QIAamp DNA FFPE kits (Qiagen, Germantown, Maryland, USA) following standard protocols. A sufficient amount of DNA (over 1 μg) was extracted from 18 primary tumors, 40 matched metastatic LNs, and adjacent normal tissue. Each sample was prepared according to Agilent library preparation protocols (Agilent SureSelect Human All exon V6 kit, Agilent, Santa Clara, CA, USA). Libraries underwent paired‐end sequencing on an Illumina HiSeq 4000 instrument according to the manufacturer's protocol.

### 2.3 Bioinformatics analyses of somatic mutations

To generate analysis‐ready bam files from Fastq files, we followed the ‘Best Practices’ workflow suggested by the Broad Institute. Briefly, raw sequencing files were aligned to the Genome Research Consortium human build 38 (GRCh38) using bwa‐mem [13]. After applying MarkDuplicates and Base Recalibration processes, initial raw candidate variants were called for each sample using the mutect2 caller [14, 15]. To reduce false‐positive variants, all variants were filtered using the following in‐house filtering criteria: (a) total depth < 50, (b) variant allelic frequency < 7%, (c) altered read counts < 4. Additional filtering criteria were then applied to assess pathogenicity: (a) combined annotation dependent depletion (CADD) phred score < 26, and (b) minor allele frequency (MAF) > 0.1% in the gnomAD database for global and East Asian populations [16]. To extract mutational signatures based on 30 recurrent base substitution patterns from the Catalog of Somatic Mutations in Cancer (cosmic), we used a public web service program called Mutalisk [17]. Copy number log‐ratios were computed with cnvkit [18]. Unsupervised clustering of cnv was performed using the r package ‘cntools’ [19]. Oncoplots were constructed using maftools [20]. Mutated genes were categorized based on oncogenic signaling pathways reported in TCGA [21]. Tumor mutational burden (TMB) was analyzed by comparison with previously reported data following basic scripts in maftools [22].

### 2.4 Immunohistochemistry

Pathologist (SJS) reviewed all hematoxylin and eosin (H&E) slides used at the time of diagnosis. Formalin‐fixed, paraffin‐embedded tissue blocks of metastatic LNs chosen, and sections (4‐μm thickness) from each metastatic LN blocks were immunostained with a primary antibody against β‐catenin (1 : 200, mouse monoclonal, Cell marque, Merck, Darmstadt, Germany), using the Ventana Benchmark XT automated staining system (Ventana Medical Systems, Tucson, AZ, USA) according to the manufacturer's protocol.

### 2.5 Bioinformatics‐based clonal analysis

To analyze ancestral sub‐clones of each metastatic LN, somatic mutations in the primary tumor were listed without considering pathogenicity (CADD phred score and MAF score). Variant allelic frequency (VAF) of selected candidates from metastatic LNs was calculated using an in‐house script. Based on these processes, it was not necessary to interpret somatic mutations in the LNs themselves during the process of LNs metastasis. Clonal lineage reconstruction and VAF‐based clustering were performed using LICHeE with all parameters set to default settings except for the following: ‐maxVAFAbsent 0.005, ‐minVAFPresent 0.05, ‐minClusterSize 5. The best‐scored lineage tree from each sample was exported in DOT format for graphviz visualization [23].

### 2.6 Statistical analysis

To determine somatic mutations associated with ENE status, we grouped somatic mutations into clusters based on the previously mentioned oncogenic signaling pathways. Categorical variables consisting of mutations occurring in each signaling pathway were compared using the Fisher's exact test. Statistical analyses were performed with spss version 26 (IBM Corp., Armonk, NY, USA), and forest plots were generated using graphpad prism version 6 (GraphPad Software, La Jolla, CA, USA).

## 3 Results

### 3.1 Tumor characteristics

**Fig. 1:**
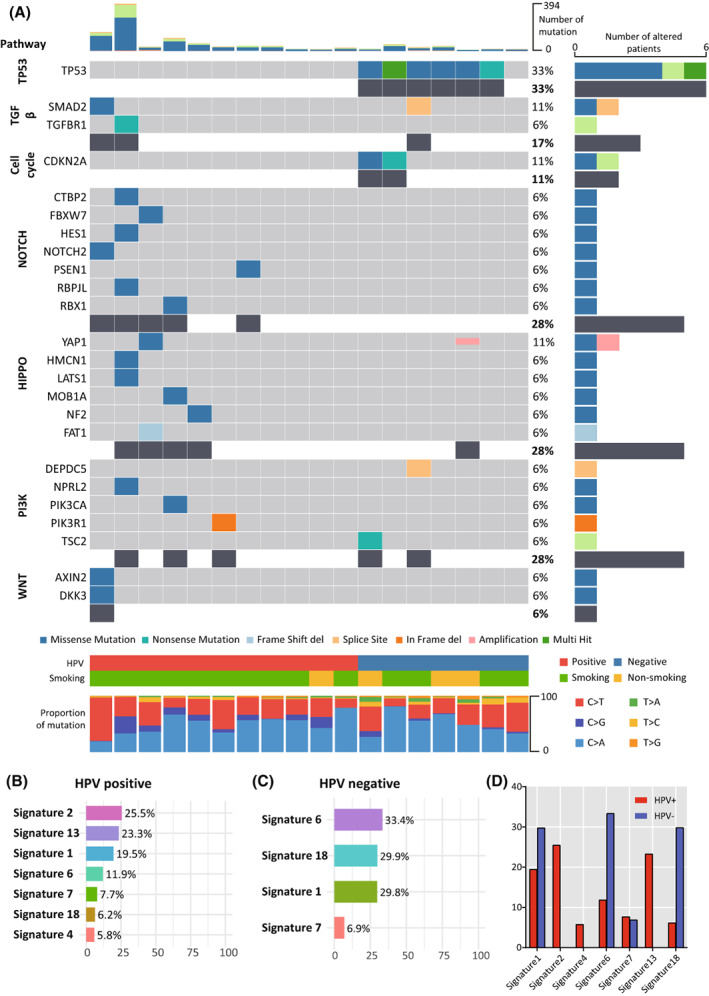
Somatic mutational profile of primary tumors. (A) Oncoplots showing mutated genes grouped based on oncogenic signaling pathways with associated characteristics. (B) Mutational signature of HPV‐positive OPSCC. (C) Mutational signature of HPV‐negative OPSCC. (D) Contribution of each mutational signature according to HPV status. HPV, human papilloma virus.

A total of 76 samples (18 from primary tumors, 40 from metastatic LNs, and 18 from normal tissue) were obtained from 18 patients with OPSCC. Among 40 metastatic LNs, 22 metastatic LNs had ENE findings (Table 1). First, we evaluated the genetic landscape of the primary tumors according to HPV status (Fig. 1A). Somatic mutations were categorized based on oncogenic signaling pathways previously reported in TCGA. Similar to previous studies, HPV‐negative OPSCC samples were characterized by somatic mutations in a cell cycle gene (*CDKN2A*) and *TP53*. Multiple somatic mutations in Notch, hippo, and WNT pathways were observed only in HPV‐positive OPSCC. Somatic mutations related to the PI3K pathway were observed in both groups. As most of the samples with high TMB were HPV‐positive, more mutations were detected in HPV‐positive patient samples. Mutational signatures were analyzed based on 30 recurrent base substitution patterns from the Catalog of Somatic Mutations in Cancer (cosmic). As previous reports indicated, HPV‐positive OPSCC showed enrichment of signatures related to APOBEC (signatures 2 and 13). In the HPV‐positive patients, 90.9% (10 out of 11) had a history of smoking, and in the HPV‐negative patients, 57.1% (4 out of 7) had a history of smoking. As expected, HPV‐positive patient samples had more smoking‐associated signatures (signatures 1, 2, 4, 5, and 13; Fig. 1B) [24]. While HPV‐negative OPSCC presented with enrichment of signatures 6, 18, and 1 (defective mismatch repair, damage of reactive oxygen species, and spontaneous deamination, respectively; Fig. 1C,D). The distribution of TMB among our samples was similar to that reported previously for TCGA data. The most common somatic mutations were single nucleotide polymorphisms (SNPs), with missense mutations the most common type of SNP (Fig. S1). Copy number variation (CNV) analysis showed a gain of 3q and 8q and loss of 3p, 11, and 13. Unsupervised clustering analysis showed no distinctive subsets according to HPV status (Fig. S2).

**Table 1:** Tumor sample characteristics. When multiple metastatic lymph node samples were collected from one patient, they were collected from different neck levels (level II–IV). LN, lymph node; pN, pathologic N stage; pT, pathologic T stage.

ID #	Age	Sex	pT	pN	Smoking history	P16 status	Tumor samples	LNs without ENE	LNs with ENE
HN01	66	M	2	2	O	Positive	1	2	1
HN02	64	M	2	1	O	Positive	1	1	1
HN03	80	M	2	2	O	Positive	1	2	0
HN04	64	M	1	1	O	Positive	1	1	1
HN05	64	M	2	2	X	Positive	1	0	2
HN06	72	M	2	2	O	Positive	1	1	2
HN07	57	M	3	2	O	Positive	1	0	3
HN08	72	M	2	2	O	Positive	1	0	3
HN09	66	M	2	2	O	Positive	1	0	1
HN10	61	M	2	2	O	Positive	1	0	3
HN11	64	M	2	1	O	Positive	1	1	1
HN12	59	M	2	2b	O	Negative	1	0	1
HN13	60	F	2	2b	X	Negative	1	1	0
HN14	63	F	3	3b	X	Negative	1	2	1
HN15	45	F	4	3b	X	Negative	1	1	1
HN16	28	M	3	3b	O	Negative	1	1	1
HN17	67	M	4	3b	O	Negative	1	3	0
HN18	59	M	4	3b	O	Negative	1	0	2

**Fig. 2:**
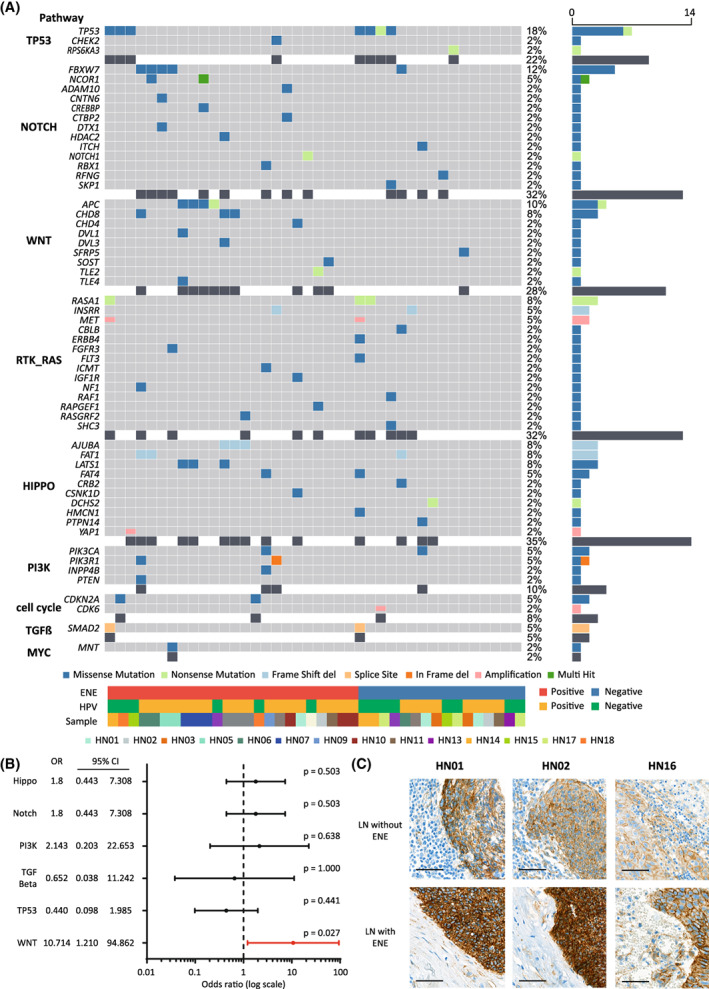
Genetic analysis of metastatic LNs. (A) Oncoplot representing mutations grouped based on oncogenic signaling pathways with associated characteristics. (B) Forest plot showing odds ratios and confidence intervals of ENE findings in association with mutated pathways(Fisher's exact test). Odds, odds ratio; *P*, *P*‐value. (C) Representative immunohistochemical staining of β‐catenin. Metastatic LNs with ENE showed strong intensity staining, but metastatic LN without ENE showed moderate intensity β‐catenin staining. Upper panels show metastatic LNs without ENE, lower panels show metastatic LNs with ENE. Scale bar = 200 micrometer; ENE, extranodal extension; HPV, human papilloma virus; LN, lymph node.

To analyze when acquired or missing somatic mutations occurred during the process of LN metastasis, we generated oncoplots representing the mutational profiles of all primary tumor and metastatic LN samples (Fig. S3). Interestingly, metastatic LNs had unique somatic mutations compared with their primary tumor and other metastatic LNs located at different neck levels in the same patient. To evaluate pathogenic somatic mutations related to ENE of metastatic LNs, we analyzed the somatic mutations frequently occurred in the metastatic LN with ENE. Surprisingly, somatic mutations of WNT pathway were observed in 41.7% (10 out of 24) from metastatic LNs with ENE compared with 6.3% (1 out of 16) from metastatic LNs without ENE and 5.6% (1 out of 18) from the primary tumor. In addition, most mutations (90.9%, 10 out of 11) were observed in HPV‐positive patients (Fig. 2A). Among metastatic LNs with ENE, two somatic mutations were nonsense mutations in tumor suppressor genes (*TLE2* and *APC*) while 12 somatic mutations (*SFRP5*, *CHD4*, *CHD8*, *DVL1*, *APC*, *TLE4*, and *SOST*) were missense mutations that had a probable or possibly damaging effect based on PolyPhen prediction scores. Additionally, all somatic missense mutations had a phred score over 27 CADD (Table S1). To determine the correlation between mutations in oncogenic signaling pathways and ENE findings, we performed the statistical analysis. Somatic mutations in the WNT pathway were significantly associated with ENE based on a two‐tailed Fisher's exact test (odds ratio = 10.714, 95% confidence interval = 1.210–94.862, *P* = 0.027, Fig. 2B). To determine whether metastatic LNs containing WNT pathway mutations resulted in up‐regulation of the WNT pathway, we stained metastatic LNs with an antibody specific to β‐catenin to determine activation of the WNT pathway. As the expression of β‐catenin may be increased in cancer cells, we compared the intensity of β‐catenin staining between metastatic LNs with and without ENE. Compared with metastatic LNs without ENE, metastatic LNs with ENE showed more intense β‐catenin staining than metastatic LNs without ENE (Fig. 2C).

**Fig. 3:**
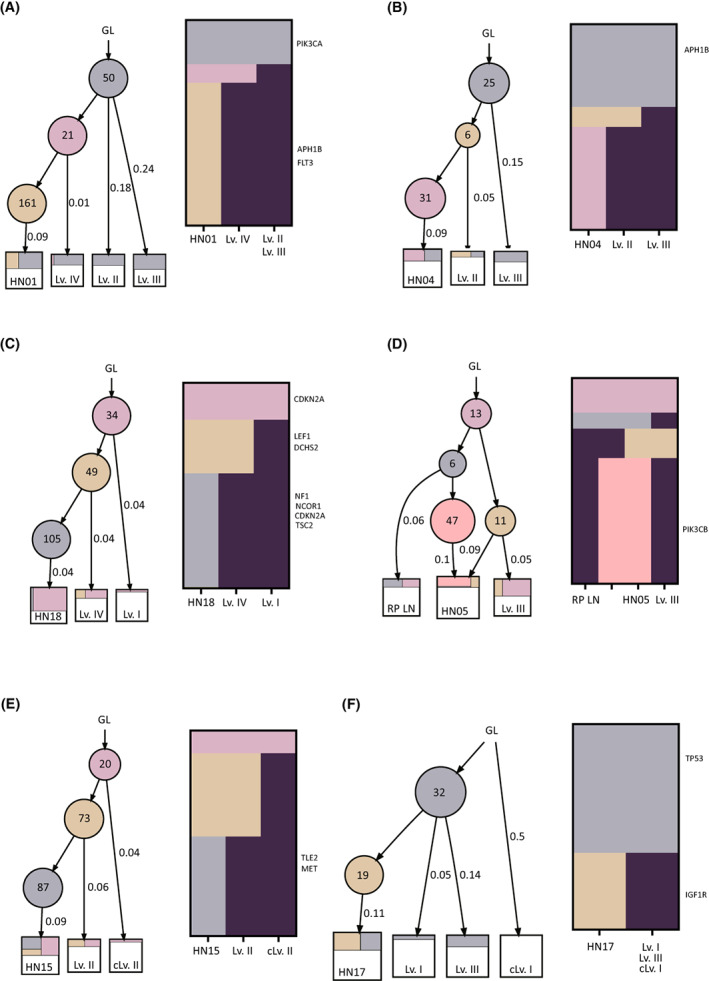
Phylogenetic trees of the primary tumor and metastatic LNs. Each circle represented a sub‐clone of the primary tumor. The number of somatic mutations is indicated in circles. The mean variant allelic fractions of sub‐clones are indicated next to the line. Heatmap nearby each tree showed the presence of each mutation in each sub‐clone. Columns in the heatmap represented each sub‐clone. Right label of heatmap showed somatic mutations included in the oncogenic signaling pathway reported in the previous report. (A) Level IV metastatic LNs of HN01 originated from different sub‐clones than Level II and III metastatic LNs. (B) Level II metastatic LNs of HN04 were derived from ancestral sub‐clones rather than Level III metastatic LNs. (C) Level IV metastatic LNs of HN18 were derived from different sub‐clones than Level I metastatic LNs. (D) Retropharyngeal metastatic LNs of HN05 were derived from different sub‐clones than level III metastatic LNs. (E) Contralateral level II metastatic LNs of HN15 originated from an ancestral sub‐clone rather than level II metastatic LNs. (F) Contralateral level I metastatic LNs of HN17 originated from an ancestral sub‐clone rather than level I and II metastatic LNs. cLv, contralateral level; GL, germline; Lv, level; RP, retropharyngeal.

Finally, we evaluated the clonal evolution of primary tumors and metastatic LNs. We harvested metastatic tissues from LNs at different neck levels. By analyzing the initial sub‐clones of each metastatic LN, we were able to infer the evolutionary process of LN metastases. We extracted the VAF of somatic mutations belonging to primary tumors only for each metastatic LN. By evaluating only the somatic mutations of primary tumors, we could infer the initial sub‐clones responsible for each metastatic LN. If metastatic LNs spread sequentially along the jugular lymph node chain from upper neck levels down to lower levels, all metastatic LNs should originate from the same sub‐clone of the primary tumor. We found that most metastatic LNs originated from the same sub‐clone of the primary tumor (Fig. S4). However, several metastatic LNs did not originate from the same sub‐clone of the primary tumor, i.e., they arose independently from different sub‐clones of the primary tumor. For example, level IV metastatic LNs of sample HN01 originated from different sub‐clone of the primary tumor. Level III metastatic LNs of sample HN04 were derived from a more ancestral sub‐clone than level II metastatic LNs. Level IV metastatic LNs of sample HN18 originated from independent sub‐clones different from the sub‐clone from which level I metastatic LNs arose. These different origins of metastatic LNs mean that LN metastases can arise directly from the primary tumor rather than from other metastatic LNs located at upper LN levels. Interestingly, we observed that contralateral metastatic LNs and retropharyngeal LNs originated from sub‐clones that were completely different to those from which ipsilateral metastatic LNs arose in samples HN05, HN15, and HN17. Taken together, our results suggest that cancer cells of metastatic LNs not only come from ipsilateral metastatic LNs but also from the primary tumor (Fig. 3).

## 4 Discussion

Here, using multiregional whole‐exome sequencing of the matched primary tumor and metastatic LN samples, we analyzed the genetic landscape and clonal evolution of primary tumors and metastatic LNs in HPV‐positive and HPV‐negative OPSCC. First, we analyzed the somatic mutational profile of the primary tumors of OPSCC patients and observed similar patterns of somatic mutations as reported previously. We found that acquired somatic mutations in the WNT pathway showed a significant association with ENE of metastatic LNs and that the WNT pathway activity was up‐regulated in metastatic LNs with ENE. Finally, we confirmed that metastatic LNs not only originated from sub‐clones of other ipsilateral metastatic LNs located at different neck levels but that some also arose from independent sub‐clones of the primary tumor.

HPV status has received attention recently as an important prognostic factor in determining treatment outcomes of OPSCC patients. Treatment results are better for HPV‐positive OPSCC patients than HPV‐negative OPSCC patients in terms of disease‐free survival (DFS), tumor‐specific survival (TSS), and overall survival (OS) [25]. Therefore, the 8th American Joint Committee on Cancer (AJCC) staging system modified the stage of OPSCC to reflect the prognostic implication of p16+ as representative of HPV status [26]. As expected, the somatic mutations, mutational signatures, and copy number alterations that we detected in primary tumors of OPSCC were similar to those found previously [21, 27, 28, 29]. Because our analysis was based on Asians, there may be limitations in interpreting the result. However, we detected a smaller overall number of somatic mutations in HPV‐negative OPSCC samples than in HPV‐positive OPSCC samples, and somatic mutations in NOTCH and HIPPO pathways were detected only in HPV‐positive OPSCC samples, but these findings should be interpreted with caution because of the limited number of HPV‐negative OPSCC samples analyzed in this study. To overcome the low quality of DNA extracted from FFPE samples, we applied stringent criteria to reduce the number of false‐positive variants. We found that mutations present in some high‐purity samples had a great effect on the overall genetic landscape. Notwithstanding, our finding that the most frequently detected mutations in HPV‐negative OPSCC samples were in *CDKN2A* and *TP53* is consistent with previous studies. These results indirectly indicate that our analysis based on FFPE samples was not distorted by the limited number of samples or unbalanced sample quality.

Lymph node metastasis itself is considered an important feature in cancer treatment and prognosis evaluation and is believed to be an intermediate step in the progression of cancer during which distant metastasis occurs [30, 31, 32]. Moreover, ENE has long been considered a pathologic high‐risk feature, meaning that patients with the finding of ENE are at an increased risk of disease progression and would benefit from adjuvant therapy [33]. Although the pathologic LN status of HPV‐positive OPSCC is based solely on the number of positive lymph nodes in the revised 8th AJCC staging system, the finding of ENE is still considered an important risk factor when planning adjuvant treatment. In addition, several studies have reported that ENE is significantly related to poorer DFS, PFS, and OS even in HPV‐positive OPSCC patients [34, 35]. In this study, we found that acquired somatic mutations in the WNT pathway were significantly associated with ENE of metastatic LNs. Furthermore, we demonstrated that the WNT pathway was activated in metastatic LNs with ENE compared to LNs without ENE in the same patients using immunohistochemical staining of β‐catenin as a biomarker of WNT pathway activation. The previous study reported an association between ENE in metastatic LNs with *CTTN* and *MMP9* mutations [36]. In addition, *CTTN* and *MMP9* are related to the WNT activation [37, 38, 39]. Due to the limited number of samples that we evaluated, our results need to be validated in a larger cohort. Nevertheless, the association between ENE and somatic mutations in the WNT pathway suggests another therapeutic approach to improve refractory OPSCC based on the control of locoregional recurrences associated with ENE of metastatic LNs.

OPSCC metastasizes to first echelon LNs according to the location of the primary tumor, then sequentially spreads to ipsilateral LNs [40, 41]. As OPSCC commonly metastasizes to level II LNs followed by level III‐IV LNs irrespective of HPV status, elective neck dissection including that of level II–IV LNs is usually performed in the surgical treatment of N0 OPSCC patients. Several studies have demonstrated that the number and location of metastatic LNs are critical predictors of treatment outcomes and survival, but studies on why these nodal factors of metastatic LNs affect prognosis are lacking [30, 42, 43, 44]. In the current study, we found that a large number of somatic mutations occurred in metastatic LNs. We compared clonal evolution between primary tumor and metastatic LN samples and found that metastatic LNs not only originated from ipsilateral metastatic LNs located at different neck levels but that some also arose independently from sub‐clones of the primary tumor. Since we applied strict filtering criteria to reduce false‐positive calls, it was thought that there were many false‐negative mutations in metastatic LNs. Therefore, we set the somatic mutations identified in the primary tumor as the correct mutation set, and the presence or absence of mutations was checked in each metastatic LN. We could demonstrate the heterogeneity of the primary tumor, but it was difficult to infer the heterogeneity of each metastatic LNs itself. However, these findings suggest the presence of inter‐lesional heterogeneity in metastatic LNs at different neck levels in OPSCC patients. We attempted to evaluate which oncogenic signaling pathway affects phylogenetic tree separation but did not find a clear correlation. This may be due to the low number of the oncogenic signaling pathway involved. Previous studies have demonstrated that a higher level of intratumoral heterogeneity is related to a poorer prognosis [45, 46, 47]. Although further research is required, inter‐lesional heterogeneity of metastatic LNs in OPSCC may lead to a poor prognosis and treatment resistance, as does intratumoral heterogeneity of the primary tumor. Furthermore, our analysis suggested that in order to plan the targeted therapy, mutation analysis should be performed in consideration of the location of recurrence, not only the genetic analysis of the primary tumor.

## 5 Conclusions

Somatic mutations in WNT pathway‐associated genes were significantly associated with ENE of metastatic LNs, and WNT pathway activity was up‐regulated in metastatic LNs. Furthermore, some metastatic LNs, including contralateral or retropharyngeal LNs, were found to originate independently from sub‐clones of the primary tumor rather than from ipsilateral metastatic LNs located elsewhere in the neck. Future studies should investigate how inter‐lesional heterogeneity of metastatic LNs of OPSCC patients affects treatment outcomes and prognosis.

## Conflict of interest

The authors declare no conflict of interest.

## Author contribution

YMP and S‐HK designed the study. NSS, S‐JS, IP, SOY, and YWK collected or analyzed the data. NSS and S‐JS performed the experiments. NSS, S‐JS, and YMP constructed the figures and wrote the manuscript. All authors provided critical manuscript editing and feedback.

### Peer review

The peer review history for this article is available at https://publons.com/publon/10.1002/1878‐0261.13407.

## Supporting information


**Table S1.** Somatic mutations from metastatic LNs in WNT pathway.
**Fig. S1.** Summary of somatic mutations in primary tumors.
**Fig. S2.** Copy number analysis of primary tumor.
**Fig. S3.** Oncoplots showing somatic mutations from all sample.
**Fig. S4.** Phylogenetic tree from a typical metastasis model.

## Data Availability

All data generated in this study have been deposited into the National Center for Biotechnology Information Sequence Read Archive with accession number PRJNA881796 (https://www.ncbi.nlm.nih.gov/sra).

## References

[1] Ang KK , Harris J , Wheeler R , Weber R , Rosenthal DI , Nguyen‐Tan PF , et al. Human papillomavirus and survival of patients with oropharyngeal cancer. N Engl J Med. 2010;363:24–35. 10.1056/NEJMoa0912217 20530316 PMC2943767

[2] Fakhry C , Zhang Q , Gillison ML , Nguyen‐Tan PF , Rosenthal DI , Weber RS , et al. Validation of NRG oncology/RTOG‐0129 risk groups for HPV‐positive and HPV‐negative oropharyngeal squamous cell cancer: implications for risk‐based therapeutic intensity trials. Cancer. 2019;125:2027–38. 10.1002/cncr.32025 30913305 PMC6594017

[3] Rosenthal DI , Harari PM , Giralt J , Bell D , Raben D , Liu J , et al. Association of Human Papillomavirus and p16 status with outcomes in the IMCL‐9815 phase III registration trial for patients with Locoregionally advanced oropharyngeal squamous cell carcinoma of the head and neck treated with radiotherapy with or without Cetuximab. J Clin Oncol. 2016;34:1300–8. 10.1200/JCO.2015.62.5970 26712222 PMC5070577

[4] Chen AM , Felix C , Wang PC , Hsu S , Basehart V , Garst J , et al. Reduced‐dose radiotherapy for human papillomavirus‐associated squamous‐cell carcinoma of the oropharynx: a single‐arm, phase 2 study. Lancet Oncol. 2017;18:803–11. 10.1016/S1470-2045(17)30246-2 28434660 PMC6488353

[5] Chera BS , Amdur RJ , Tepper JE , Tan X , Weiss J , Grilley‐Olson JE , et al. Mature results of a prospective study of deintensified chemoradiotherapy for low‐risk human papillomavirus‐associated oropharyngeal squamous cell carcinoma. Cancer. 2018;124:2347–54. 10.1002/cncr.31338 29579339

[6] Gillison ML , Trotti AM , Harris J , Eisbruch A , Harari PM , Adelstein DJ , et al. Radiotherapy plus cetuximab or cisplatin in human papillomavirus‐positive oropharyngeal cancer (NRG oncology RTOG 1016): a randomised, multicentre, non‐inferiority trial. Lancet. 2019;393:40–50. 10.1016/S0140-6736(18)32779-X 30449625 PMC6541928

[7] Marur S , Li S , Cmelak AJ , Gillison ML , Zhao WJ , Ferris RL , et al. E1308: phase II trial of induction chemotherapy followed by reduced‐dose radiation and weekly Cetuximab in patients with HPV‐associated Resectable squamous cell carcinoma of the oropharynx‐ ECOG‐ACRIN Cancer research group. J Clin Oncol. 2017;35:490–7. 10.1200/JCO.2016.68.3300 28029303 PMC5455313

[8] Stock GT , Bonadio R , de Castro GJ . De‐escalation treatment of human papillomavirus‐positive oropharyngeal squamous cell carcinoma: an evidence‐based review for the locally advanced disease. Curr Opin Oncol. 2018;30:146–51. 10.1097/CCO.0000000000000441 29474271

[9] Chotchutipan T , Rosen BS , Hawkins PG , Lee JY , Saripalli AL , Thakkar D , et al. Volumetric (18) F‐FDG‐PET parameters as predictors of locoregional failure in low‐risk HPV‐related oropharyngeal cancer after definitive chemoradiation therapy. Head Neck. 2019;41:366–73. 10.1002/hed.25505 30548704 PMC6411288

[10] Hawkins PG , Mierzwa ML , Bellile E , Jackson WC , Malloy KM , Chinn SB , et al. Impact of American joint committee on Cancer eighth edition clinical stage and smoking history on oncologic outcomes in human papillomavirus‐associated oropharyngeal squamous cell carcinoma. Head Neck. 2019;41:857–64. 10.1002/hed.25336 30775826 PMC6420360

[11] Lydiatt WM , Patel SG , O'Sullivan B , Brandwein MS , Ridge JA , Migliacci JC , et al. Head and neck cancers‐major changes in the American joint committee on cancer eighth edition cancer staging manual. CA Cancer J Clin. 2017;67:122–37. 10.3322/caac.21389 28128848

[12] O'Sullivan B , Huang SH , Su J , Garden AS , Sturgis EM , Dahlstrom K , et al. Development and validation of a staging system for HPV‐related oropharyngeal cancer by the international collaboration on oropharyngeal cancer network for staging (ICON‐S): a multicentre cohort study. Lancet Oncol. 2016;17:440–51. 10.1016/S1470-2045(15)00560-4 26936027

[13] McKenna A , Hanna M , Banks E , Sivachenko A , Cibulskis K , Kernytsky A , et al. The Genome analysis toolkit: a MapReduce framework for analyzing next‐generation DNA sequencing data. Genome Res. 2010;20:1297–303. 10.1101/gr.107524.110 20644199 PMC2928508

[14] Cibulskis K , Lawrence MS , Carter SL , Sivachenko A , Jaffe D , Sougnez C , et al. Sensitive detection of somatic point mutations in impure and heterogeneous cancer samples. Nat Biotechnol. 2013;31:213–9. 10.1038/nbt.2514 23396013 PMC3833702

[15] DePristo MA , Banks E , Poplin R , Garimella KV , Maguire JR , Hartl C , et al. A framework for variation discovery and genotyping using next‐generation DNA sequencing data. Nat Genet. 2011;43:491–8. 10.1038/ng.806 21478889 PMC3083463

[16] Karczewski KJ , Francioli LC , Tiao G , Cummings BB , Alfoldi J , Wang Q , et al. The mutational constraint spectrum quantified from variation in 141,456 humans. Nature. 2020;581:434–43. 10.1038/s41586-020-2308-7 32461654 PMC7334197

[17] Lee J , Lee AJ , Lee JK , Park J , Kwon Y , Park S , et al. Mutalisk: a web‐based somatic MUTation AnaLyIS toolKit for genomic, transcriptional and epigenomic signatures. Nucleic Acids Res. 2018;46:W102–8. 10.1093/nar/gky406 29790943 PMC6030918

[18] Talevich E , Shain AH , Botton T , Bastian BC . CNVkit: Genome‐wide copy number detection and visualization from targeted DNA sequencing. PLoS Comput Biol. 2016;12:e1004873. 10.1371/journal.pcbi.1004873 27100738 PMC4839673

[19] Zhang J . CNTools: convert segment data into a region by sample matrix to allow for other high level computational analyses. 2020.

[20] Mayakonda A , Lin DC , Assenov Y , Plass C , Koeffler HP . Maftools: efficient and comprehensive analysis of somatic variants in cancer. Genome Res. 2018;28:1747–56. 10.1101/gr.239244.118 30341162 PMC6211645

[21] Sanchez‐Vega F , Mina M , Armenia J , Chatila WK , Luna A , La KC , et al. Oncogenic signaling pathways in the Cancer Genome Atlas. Cell. 2018;173:321–37 e310. 10.1016/j.cell.2018.03.035 29625050 PMC6070353

[22] Ellrott K , Bailey MH , Saksena G , Covington KR , Kandoth C , Stewart C , et al. Scalable Open Science approach for mutation calling of tumor exomes using multiple genomic pipelines. Cell Syst. 2018;6:271–81.e7. 10.1016/j.cels.2018.03.002 29596782 PMC6075717

[23] Popic V , Salari R , Hajirasouliha I , Kashef‐Haghighi D , West RB , Batzoglou S . Fast and scalable inference of multi‐sample cancer lineages. Genome Biol. 2015;16:91. 10.1186/s13059-015-0647-8 25944252 PMC4501097

[24] Alexandrov LB , Ju YS , Haase K , Van Loo P , Martincorena I , Nik‐Zainal S , et al. Mutational signatures associated with tobacco smoking in human cancer. Science. 2016;354:618–22. 10.1126/science.aag0299 27811275 PMC6141049

[25] D'Souza G , Kreimer AR , Viscidi R , Pawlita M , Fakhry C , Koch WM , et al. Case‐control study of human papillomavirus and oropharyngeal cancer. N Engl J Med. 2007;356:1944–56. 10.1056/NEJMoa065497 17494927

[26] Amin MB , Greene FL , Edge SB , Compton CC , Gershenwald JE , Brookland RK , et al. The eighth edition AJCC Cancer staging manual: continuing to build a bridge from a population‐based to a more "personalized" approach to cancer staging. CA Cancer J Clin. 2017;67:93–9. 10.3322/caac.21388 28094848

[27] Cancer Genome Atlas Network . Comprehensive genomic characterization of head and neck squamous cell carcinomas. Nature. 2015;517:576–82. 10.1038/nature14129 25631445 PMC4311405

[28] Gillison ML , Akagi K , Xiao W , Jiang B , Pickard RKL , Li J , et al. Human papillomavirus and the landscape of secondary genetic alterations in oral cancers. Genome Res. 2019;29:1–17. 10.1101/gr.241141.118 30563911 PMC6314162

[29] Morris LGT , Chandramohan R , West L , Zehir A , Chakravarty D , Pfister DG , et al. The molecular landscape of recurrent and metastatic head and neck cancers: insights from a precision oncology sequencing platform. JAMA Oncol. 2017;3:244–55. 10.1001/jamaoncol.2016.1790 27442865 PMC5253129

[30] Ho AS , Kim S , Tighiouart M , Gudino C , Mita A , Scher KS , et al. Metastatic lymph node burden and survival in Oral cavity Cancer. J Clin Oncol. 2017;35:3601–9. 10.1200/JCO.2016.71.1176 28880746 PMC5791830

[31] Huang L , David O , Cabay RJ , Valyi‐Nagy K , Macias V , Zhong R , et al. Molecular classification of lymph node metastases subtypes predict for survival in head and neck Cancer. Clin Cancer Res. 2019;25:1795–808. 10.1158/1078-0432.CCR-18-1884 30573692 PMC6420850

[32] Pereira ER , Kedrin D , Seano G , Gautier O , Meijer EFJ , Jones D , et al. Lymph node metastases can invade local blood vessels, exit the node, and colonize distant organs in mice. Science. 2018;359:1403–7. 10.1126/science.aal3622 29567713 PMC6002772

[33] Huang SH , Chernock R , O'Sullivan B , Fakhry C . Assessment criteria and clinical implications of Extranodal extension in head and neck Cancer. Am Soc Clin Oncol Educ Book. 2021;41:265–78. 10.1200/EDBK_320939 34010048

[34] Freitag J , Wald T , Kuhnt T , Gradistanac T , Kolb M , Dietz A , et al. Extracapsular extension of neck nodes and absence of human papillomavirus 16‐DNA are predictors of impaired survival in p16‐positive oropharyngeal squamous cell carcinoma. Cancer. 2020;126:1856–72. 10.1002/cncr.32667 32032442

[35] Shevach J , Bossert A , Bakst RL , Liu J , Misiukiewicz K , Beyda J , et al. Extracapsular extension is associated with worse distant control and progression‐free survival in patients with lymph node‐positive human papillomavirus‐related oropharyngeal carcinoma. Oral Oncol. 2017;74:56–61. 10.1016/j.oraloncology.2017.09.014 29103752

[36] Zhou X , Temam S , Oh M , Pungpravat N , Huang BL , Mao L , et al. Global expression‐based classification of lymph node metastasis and extracapsular spread of oral tongue squamous cell carcinoma. Neoplasia. 2006;8:925–32. 10.1593/neo.06430 17132224 PMC1716013

[37] Ingraham CA , Park GC , Makarenkova HP , Crossin KL . Matrix metalloproteinase (MMP)‐9 induced by Wnt signaling increases the proliferation and migration of embryonic neural stem cells at low O2 levels. J Biol Chem. 2011;286:17649–57. 10.1074/jbc.M111.229427 21460212 PMC3093840

[38] Wei CY , Zhu MX , Yang YW , Zhang PF , Yang X , Peng R , et al. Downregulation of RNF128 activates Wnt/beta‐catenin signaling to induce cellular EMT and stemness via CD44 and CTTN ubiquitination in melanoma. J Hematol Oncol. 2019;12:21. 10.1186/s13045-019-0711-z 30832692 PMC6399928

[39] Wu B , Crampton SP , Hughes CC . Wnt signaling induces matrix metalloproteinase expression and regulates T cell transmigration. Immunity. 2007;26:227–39. 10.1016/j.immuni.2006.12.007 17306568 PMC1855210

[40] Candela FC , Kothari K , Shah JP . Patterns of cervical node metastases from squamous carcinoma of the oropharynx and hypopharynx. Head Neck. 1990;12:197–203. 10.1002/hed.2880120302 2358329

[41] Shah JP , Candela FC , Poddar AK . The patterns of cervical lymph node metastases from squamous carcinoma of the oral cavity. Cancer. 1990;66:109–13. 10.1002/1097-0142(19900701)66:1<109::aid-cncr2820660120>3.0.co;2-a 2354399

[42] Divi V , Chen MM , Nussenbaum B , Rhoads KF , Sirjani DB , Holsinger FC , et al. Lymph node count from neck dissection predicts mortality in head and neck Cancer. J Clin Oncol. 2016;34:3892–7. 10.1200/JCO.2016.67.3863 27480149

[43] Kuo P , Mehra S , Sosa JA , Roman SA , Husain ZA , Burtness BA , et al. Proposing prognostic thresholds for lymph node yield in clinically lymph node‐negative and lymph node‐positive cancers of the oral cavity. Cancer. 2016;122:3624–31. 10.1002/cncr.30227 27479645

[44] Roberts TJ , Colevas AD , Hara W , Holsinger FC , Oakley‐Girvan I , Divi V . Number of positive nodes is superior to the lymph node ratio and American joint committee on Cancer N staging for the prognosis of surgically treated head and neck squamous cell carcinomas. Cancer. 2016;122:1388–97. 10.1002/cncr.29932 26969807

[45] Harbst K , Lauss M , Cirenajwis H , Isaksson K , Rosengren F , Torngren T , et al. Multiregion whole‐exome sequencing uncovers the genetic evolution and mutational heterogeneity of early‐stage metastatic melanoma. Cancer Res. 2016;76:4765–74. 10.1158/0008-5472.CAN-15-3476 27216186

[46] Oxnard GR , Thress KS , Alden RS , Lawrance R , Paweletz CP , Cantarini M , et al. Association between plasma genotyping and outcomes of treatment with Osimertinib (AZD9291) in advanced non‐small‐cell lung Cancer. J Clin Oncol. 2016;34:3375–82. 10.1200/JCO.2016.66.7162 27354477 PMC5035123

[47] Piotrowska Z , Niederst MJ , Karlovich CA , Wakelee HA , Neal JW , Mino‐Kenudson M , et al. Heterogeneity underlies the emergence of EGFRT790 wild‐type clones following treatment of T790M‐positive cancers with a third‐generation EGFR inhibitor. Cancer Discov. 2015;5:713–22. 10.1158/2159-8290.CD-15-0399 25934077 PMC4497836

