# Supplementary material for: Investigation of somatic mutation profiles and tumor evolution of primary oropharyngeal cancer and sequential lymph node metastases using multiregional whole‐exome sequencing
Source: Mol Oncol. 2023 Mar 8;17(6):981–92. doi: 10.1002/1878-0261.13407 (PMC10257422; doi:10.1002/1878-0261.13407)
Supplement: Supplementary file 1 — Table S1. Somatic mutations from metastatic LNs in WNT pathway. Fig. S1. Summary of somatic mutations in primary tumors. Fig. S2. Copy number analysis of primary tumor. Fig. S3. Oncoplots showing somatic mutations from all sample. Fig. S4. Phylogenetic tree from a typical metastasis model. [file MOL2-17-981-s001.pdf]

Investigation of somatic mutation profiles and tumor evolution of primary oropharyngeal cancer and sequential lymph node metastases using multiregional whole-exome sequencing

Nam Suk Sim, Su-Jin Shin, Inho Park, Sun Och Yoon, Yoon Woo Koh, Se-Heon Kim, Young Min Park

Supplementary Table 1. Somatic mutations from metastatic LNs of WNT pathway

Somatic mutations of WNT pathway detected in metastatic LNs. Among metastatic LNs with ENE, two somatic mutations were nonsense mutations in tumor suppressor genes, while twelve somatic mutations were missense mutations that had a probable or possibly damaging effect based on polyphen prediction scores. All somatic missense mutations had a phred score over 27 CADD.

Supplementary Figure 1. **Summary of somatic mutations in primary tumors**

(A) Distribution of tumor mutational burden (TMB) compared to 33 TCGA cohorts. (B) Distribution of variants.

Supplementary Figure 2. Copy number analysis of primary tumor

Heatmap representing relative copy number alterations of primary tumors with associated characteristics. Unsupervised analysis of CNV was performed using CNtools.

Supplementary Figure 3. Oncoplots showing somatic mutations from all sample

Oncoplots showing mutated genes in all primary tumor and metastatic LN samples with associated characteristics. LN: lymph node

Supplementary Figure 4. Phylogenetic tree from a typical metastasis model

Samples showed metastatic LNs originated from the same sub-clone of the primary tumor.

Supplementary table 1

	Sample	Hugo_Symbol	Chrom	Start_Position	End_Position	NCBI_Build	Variant	Reference	Altered	HGVSc	HGVSp	PolyPhen	CADD
**WNT pathway mutations in metastatic LN with ENE**	HN01_LN1	CHD4	chr12	6594649	6594649	GRCh38	Missense	G	T	c.2123C>A	p.Pro708Gln	probably_damaging	31
	HN02_LN1	TLE2	chr19	3002353	3002353	GRCh38	Nonsense	C	A	c.2047G>T	p.Gly683Ter		47
	HN03_LN2	SFRP5	chr10	97771704	97771704	GRCh38	Missense	C	A	c.130G>T	p.Gly44Cys	probably_damaging	27.6
	HN06_LN2	CHD8	chr14	21405388	21405388	GRCh38	Missense	G	T	c.3128C>A	p.Pro1043His	probably_damaging	27.9
	HN07_LN1	DVL1	chr1	1339362	1339362	GRCh38	Missense	C	A	c.1132G>T	p.Gly378Cys	probably_damaging	35
	HN07_LN1	APC	chr5	112754945	112754945	GRCh38	Missense	G	C	c.55G>C	p.Glu19Gln	probably_damaging	27.4
	HN07_LN1	TLE4	chr9	79709632	79709632	GRCh38	Missense	G	C	c.1273G>C	p.Asp425His	probably_damaging	29.8
	HN07_LN2	APC	chr5	112754945	112754945	GRCh38	Missense	G	C	c.55G>C	p.Glu19Gln	probably_damaging	27.4
	HN07_LN3	APC	chr5	112754945	112754945	GRCh38	Missense_	G	C	c.55G>C	p.Glu19Gln	probably_damaging	27.4
	HN08_LN1	CHD8	chr14	21395054	21395054	GRCh38	Missense	G	A	c.5248C>T	p.Arg1750Trp	probably_damaging	27.9
	HN08_LN3	CHD8	chr14	21395054	21395054	GRCh38	Missense	G	A	c.5248C>T	p.Arg1750Trp	probably_damaging	27.9
	HN08_LN3	DVL3	chr3	184167711	184167711	GRCh38	Missense	G	T	c.1330G>T	p.Gly444Cys	probably_damaging	36
	HN11_LN1	SOST	chr17	43755713	43755713	GRCh38	Missense	C	T	c.271G>A	p.Asp91Asn	possibly_damaging	31
	HN16_LN1	APC	chr5	112792473	112792473	GRCh38	Nonsense	G	T	c.673G>T	p.Glu225Ter		37

Supplementary figure 1

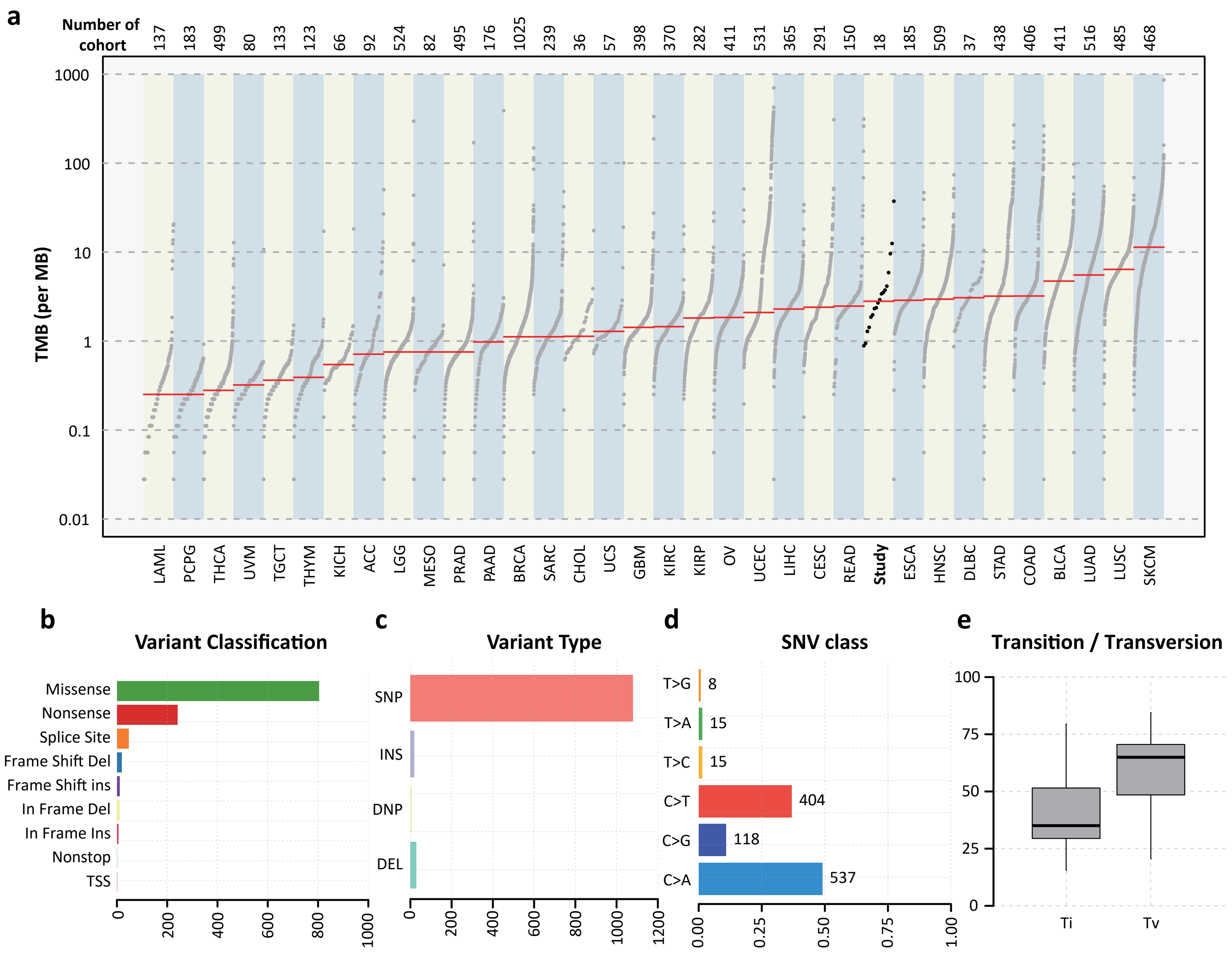

Supplementary figure 2

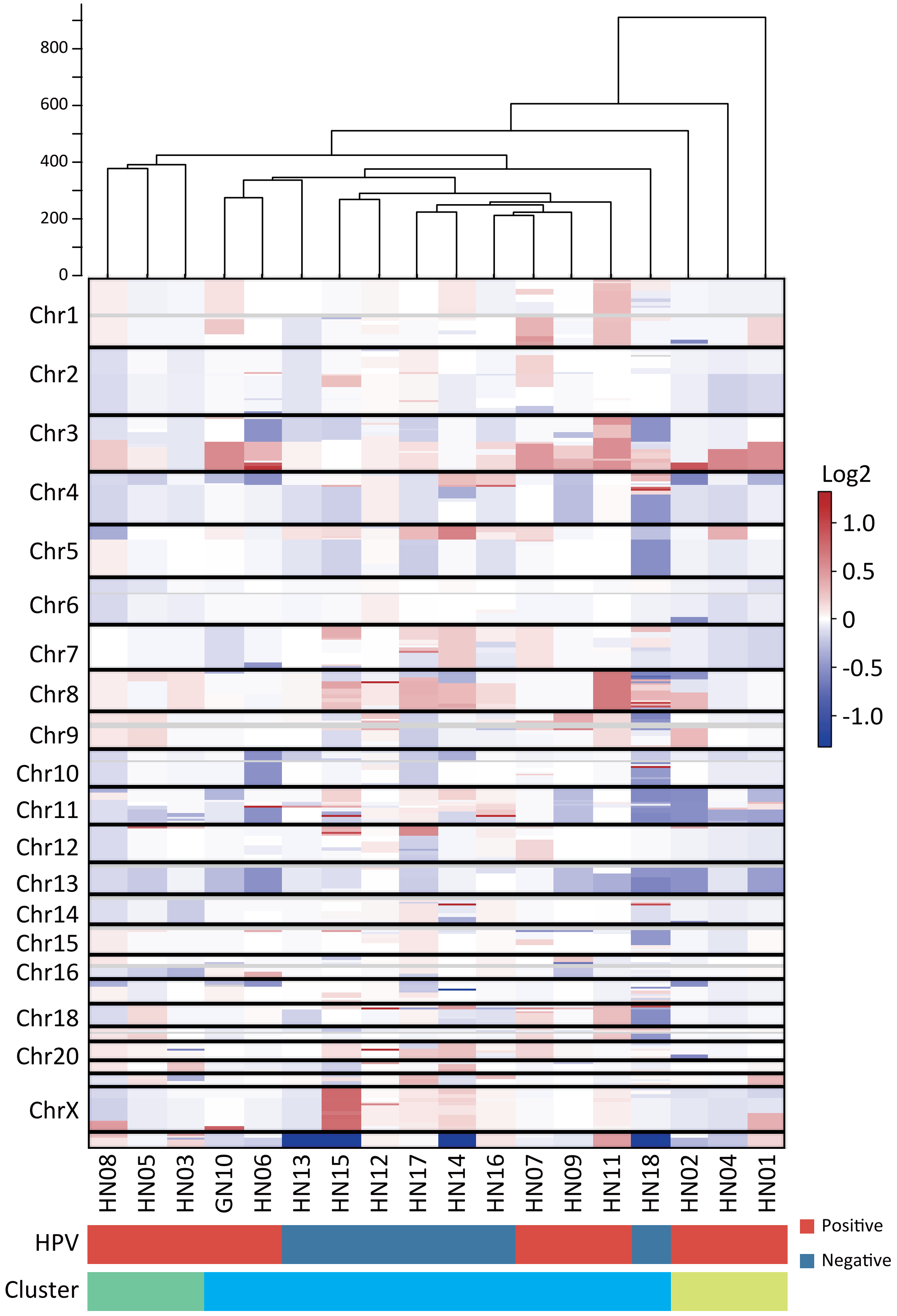

Supplementary figure 3

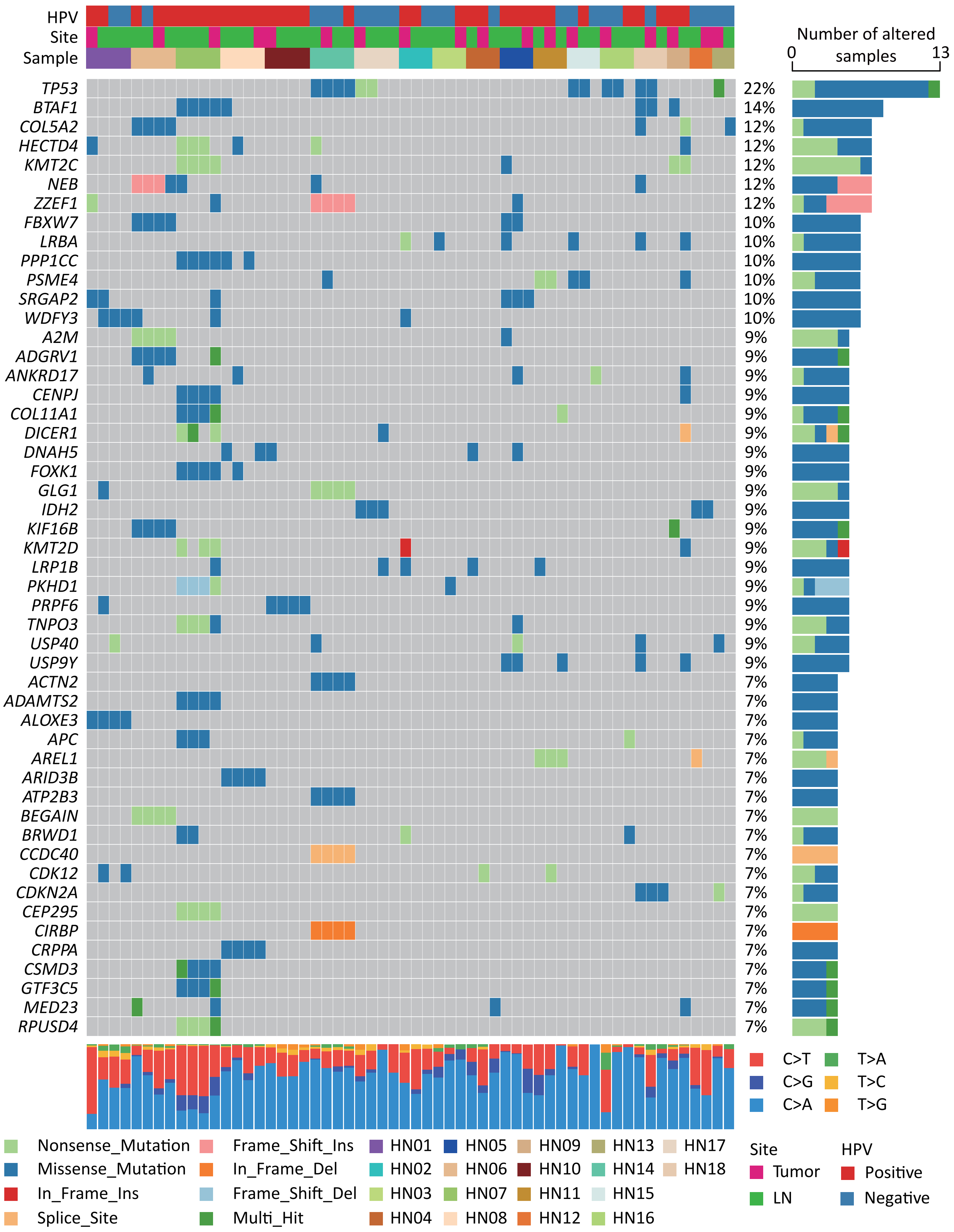

Supplementary figure 4

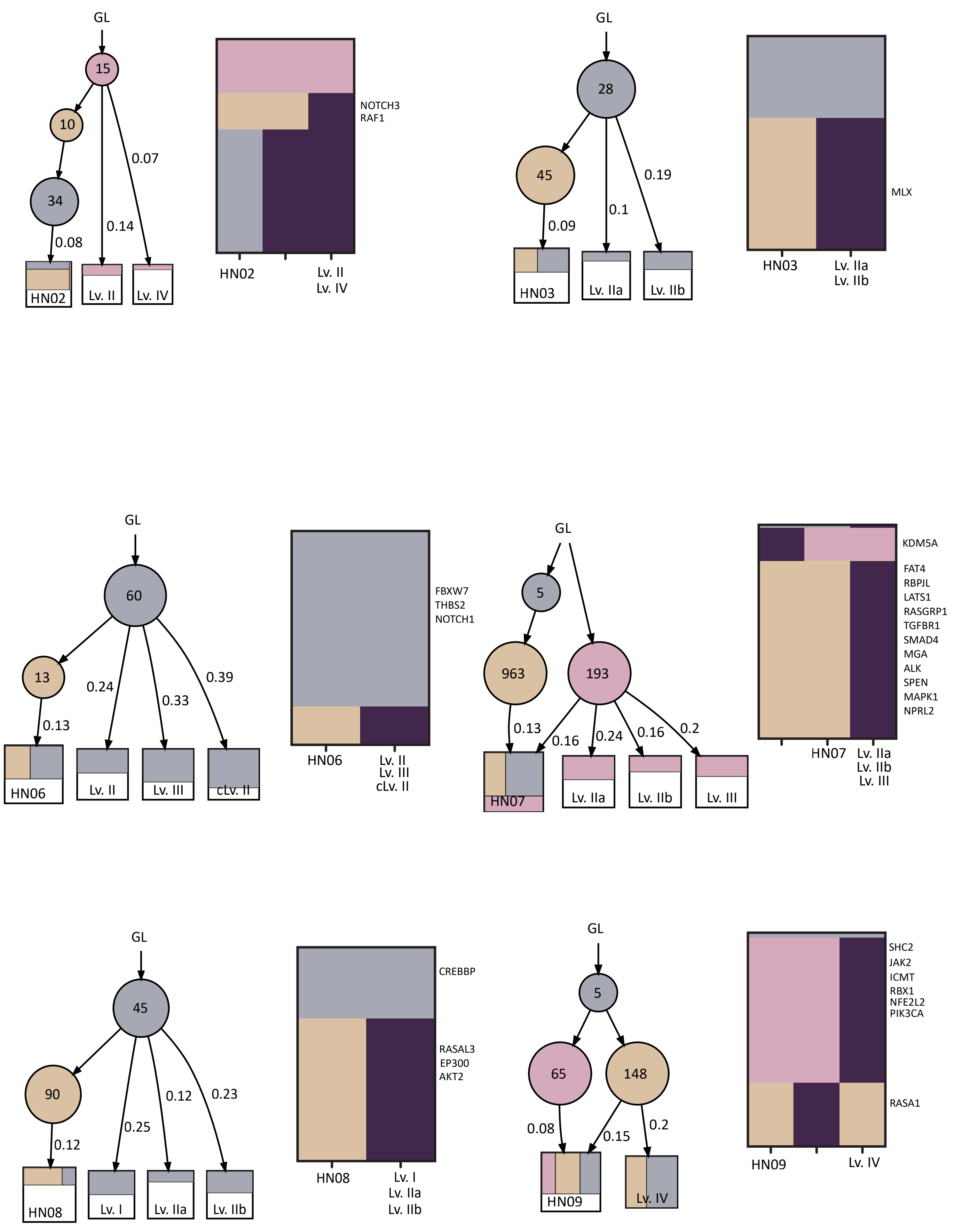

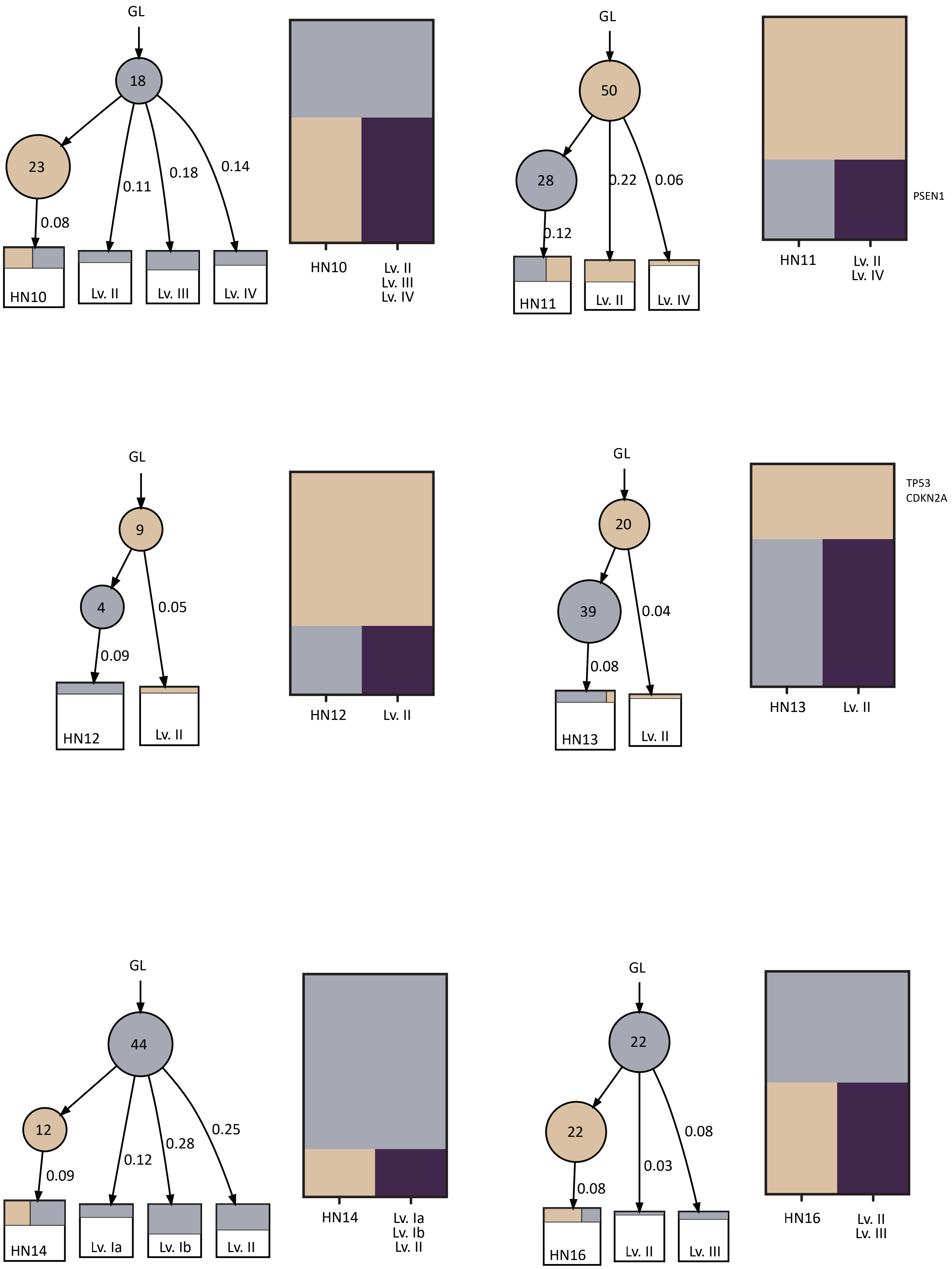
GL
18
23
0.08
0.11
0.18
0.14
HN10
Lv. II
Lv. III
Lv. IV
HN10
Lv. II
Lv. III
Lv. IV
GL
50
28
0.12
0.22
0.06
HN11
Lv. II
Lv. IV
HN11
Lv. II
Lv. IV
PSEN1
GL
9
4
0.09
0.05
HN12
Lv. II
HN12
Lv. II
GL
20
39
0.08
0.04
HN13
Lv. II
TP53
CDKN2A
HN13
Lv. II
GL
44
12
0.09
0.12
0.28
0.25
HN14
Lv. Ia
Lv. Ib
Lv. II
HN14
Lv. Ia
Lv. Ib
Lv. II
GL
22
22
0.08
0.03
0.08
HN16
Lv. II
Lv. III
HN16
Lv. II
Lv. III
